# Body Composition of Master Swimmers before and after the COVID-19 Pandemic: A Longitudinal Study

**DOI:** 10.3390/jcm12226992

**Published:** 2023-11-08

**Authors:** Grzegorz Bielec, Anna Goździejewska, Birgitta Langhammer, Krzysztof Borysławski

**Affiliations:** 1 Faculty of Physical Culture, Gdańsk University of Physical Education and Sport, 80-309 Gdańsk, Poland; 2 Department of Tourism, Recreation and Ecology, University of Warmia and Mazury, 10-719 Olsztyn, Poland; gozdzik@uwm.edu.pl; 3 Department of Physiotherapy, Oslo Metropolitan University, 0176 Oslo, Norway; birgitta.langhammer@oslomet.no; 4 Institute of Health, The Angelus Silesius University of Applied Sciences, 58-300 Wałbrzych, Poland; kboryslawski@ans.edu.pl

**Keywords:** body composition, Master athletes, swimming, COVID-19, sarcopenia

## Abstract

The long-term effect of physical activity on body composition in Master athletes is rarely presented in the literature. The aim of this study was to identify possible changes in body composition of Master swimmers over a period of 4 years, including during the COVID-19 pandemic. Additionally, we wanted to discover if sarcopenia symptoms would occur in Master athletes during the analyzed period. The body compositions of one hundred and sixty-seven Master swimmers were assessed with the InBody 270 analyzer in 2018. In 2022, the measurements were repeated in forty-six men and fourteen women. The Muscle Mass Index and the Skeletal Muscle Index were calculated to identify sarcopenia-related changes in body composition. We surveyed participants to gather data on their training patterns and SARS-CoV-2 infection history. No significant changes in body composition parameters were noted in Master swimmers during the analysis period (*p* > 0.05). Sarcopenia risk was not identified in examined athletes, and sarcopenia indicators did not change markedly over the 4-year period (*p* > 0.05). Participants with a history of SARS-CoV-2 infection did not differ from non-infected subjects in terms of body composition. Training cessation during the COVID-19 pandemic and SARS-CoV-2 infection did not induce long-term changes in body composition of Master swimmers. Life-long participation in swimming activities appears to delay negative changes in body composition, including sarcopenia symptoms.

## 1. Introduction

Physical activity (PA) is a key prerequisite for healthy aging. Specific recommendations concerning the duration and intensity of PA for various age groups are provided regularly by well-known health organizations like the World Health Organization and the American Heart Association. People who follow these recommendations are considered physically active; otherwise, they are considered sedentary. Physical activity is considered a protective factor for cardiovascular disease, stroke, and diabetes [1]. Physically active older adults are also less likely to experience a decline in mental function [2]. Older adults who undertake PA on a regular basis are less likely to be diagnosed with chronic diseases and take fewer prescription drugs than their less active counterparts [3]. Mortality can be reduced by 22% even in older adults who take part in less than the recommended amount of moderate-to-vigorous PA [4]. Regular aerobic exercises undertaken by older adults have a protective effect against sarcopenia due to muscle size and muscle strength increase, as well as reduction in myostatin mRNA expression [5].

Master athletes are typically individuals aged 40 years or older who regularly practice sports at a competitive level [6]. International sports federations specify the minimum age for Master athletes. For example, the minimum age required is 35 years old for both track and field competitions and in canoe/kayak rivalry [7,8]. Master athletes usually exercise more than 5 h per week, participating in approximately four training sessions [9]. Thus, Master athletes are characterized by much higher levels of maximal aerobic capacity and skeletal muscle metabolic fitness than their sedentary peers [10]. Master athletes also achieve higher scores in verbal memory tests and reaction time tests [11]. Master athletes and untrained peers also differ in body composition. Master strength athletes have a higher lean body mass than sedentary men of an equivalent age [12]. In another study, the body fat percentage was lower in male Master athletes than in sedentary subjects [13].

Physical activity in all age groups was significantly restricted during the COVID-19 pandemic. Indoor and outdoor sports facilities were closed due to lockdown laws, making PA inaccessible for both recreational and competitive athletes. All sports events, including the Olympic Games, were cancelled. In Europe, pandemic-related restrictions were imposed and lifted periodically between March 2020 and September 2021. Despite lockdowns, many athletes trained individually or online in reduced amounts [14]. Access to sports and recreational facilities improved over time due to growing population immunity. Both recreational and competitive athletes were able to return to their training regimes. Sports events were initially reinstated with no spectators in the stands. The ban on sports activities was ultimately lifted in the summer/autumn of 2021 [15]

According to many studies, lockdown-related training cessation exerted varied effects on athletes. The COVID-19 lockdown adversely impacted training routines for football players globally. Regardless of this fact, professional players experienced fewer disruptions compared to amateur and semi-professional players [16]. Physical fitness levels in adult elite kickboxers and football players decreased after a two-month or longer stay-at-home period [17,18]. In contrast, the first two-month lockdown in the spring of 2020 did not affect the fitness levels or energy intake of Master cyclists (mean age 47 ± 10 years) [19]. Moreover, Master athletes and lifelong exercisers were less susceptible to the SARS-CoV-2 infection due to a higher immune response [20]. In regard to body composition, a study of elite male football players (mean age 30.5 ± 3.6 years) revealed a decrease in body mass and muscle mass after the first lockdown between February and May 2020 [21]. In contrast, no distinct changes in any body composition parameters were reported in elite male fencers (mean age 26.4 ± 4.5 years) and female fencers (mean age 24.7 ± 3.8 years) between September 2019 and June 2020 [22].

Some studies reported the decline in PA of Master athletes during the COVID-19 pandemic [23]. However, the impact of lockdowns on the health and performance of Master athletes has rarely been researched. To the best of our knowledge, changes in the body composition of Master athletes during the COVID-19 pandemic have not been studied to date. At the same time, some studies have shown that COVID-19 restrictions had no significant effect on the body composition of physically inactive older adults [24,25]. On the other hand, physical inactivity, changes in nutritional habits, social isolation, and other stressful situations connected with COVID-19 lockdowns amplified the risk of sarcopenia, particularly in older adults [26,27]. Since muscle mass decreases were observed in middle-aged cohorts [28], our intention was to investigate this phenomenon among Master athletes of both genders aged 35+ in the longitudinal study. Therefore, the aim of this study was to identify changes in the body composition of Master swimmers over a period of 4 years before and after the COVID-19 pandemic. Our hypothesis exemplifies that the body composition of Master swimmers will be reduced at follow-up testing 2 years after the start of the COVID-19 pandemic. Namely, we hypothesized that fatty tissue would increase and muscle tissue would decrease during the analyzed period.

## 2. Material and Methods

### 2.1. Study Design and Participants

**Figure 1:**
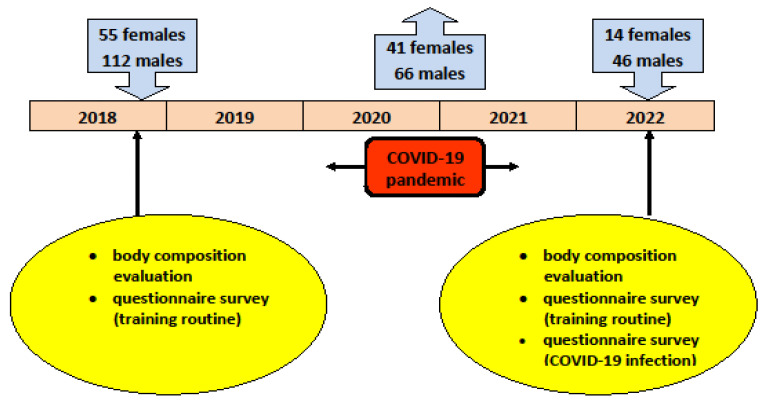
Study design and participants.

The study was conducted during the Polish Master Swimming Championships in November 2018 and June 2022. Female and male athletes participating in these events were randomly selected and invited to participate in the study. The participants were informed about the purpose of the study and the research protocol, and they signed informed consent forms. Participants older than 35 years at baseline (November 2018) were included in the study. One hundred and sixty-seven athletes (112 men with a mean age of 52.3 ± 14.5 years and 55 women with a mean age of 48.6 ± 13.6 years) were examined in November 2018. Out of those, 46 men and 14 women participated in the examination in June 2022, and their data were taken into consideration in the present study (Figure 1). The participants’ training regimes and demographic characteristics are presented in Table 1. The study has been approved by the local Institutional Review Board (No. 9/2018).

### 2.2. Data Collection

The examinations were conducted in the morning (8:30–11:00 AM) in a separate room in the indoor swimming pool. The temperature in the room was 24.6–25.2 degrees centigrade, and the humidity was set at 48–51 per cent. First, the participants completed the survey questionnaire and provided information about their age, competitive experience, training loads, and diet for the last six months before the examination [29]. Then, the participants underwent an anthropometric examination. Body height was measured with the Seca 216 stadiometer (Seca GmbH, Hamburg, Germany), and body composition was analyzed with the InBody 270 analyzer (InBody Co Ltd., Cerritos, CA, USA). InBody 270 is considered a reliable device for body composition analysis [30,31]. The participants were barefoot and wore swimsuits and t-shirts during the measurements. All measurements were performed by an experienced researcher with a PhD.

The same examination procedure (i.e., questionnaire survey and body composition assessment) was utilized during the Polish Master Swimming Championships in June 2022. The athletes surveyed in June 2022 were also queried to provide information about their history of SARS-CoV-2 infection in a standardized questionnaire developed by the Polish Ministry of Health [32]. The questionnaire included questions regarding the testing of SARS-CoV-2, hospitalization during the illness, medications taken during infection, and common symptoms of the disease.

### 2.3. Indicators

Based on the report of the European Working Group on Sarcopenia in Older People [33], the following sarcopenia indicators were calculated:

Muscle Mass Index = Total Muscle Mass/Height squared [kg/m^2^], (1)

Skeletal Muscle Index = Total Muscle Mass/Body Mass × 100 [%].(2)

The training intensity index was calculated as follows:

Training Intensity Index = Distance Covered in Training Session × Number of Training Sessions in a Week.(3)

For example, if the athletes covered an average distance of 3000 m in a single training session and had three training sessions in a week, their training intensity index was 3000 × 3 = 9000 [34].

### 2.4. Statistical Analysis

The sample size was calculated a priori with a web-based ClinCalc calculator (ClinCalc LCC, Indianapolis, IN, USA). Sample size calculations were performed based on the study of Benelli et al. [35], where 19 male Master swimmers (mean age 44.2 ± 2.5 years) and 19 female master swimmers (mean age 44.1 ± 3.6 years) were assessed in terms of body composition. Statistical significance was assumed with *p* = 0.05 and a test power value of 80%. The recommended sample size comprised 36 men and 13 women.

The Statistica for Windows software (StatSoft, Tulsa, OK, USA, version 13.1) was utilized to perform statistical calculations. For all tested parameters the Shapiro–Wilk test did not indicate significant deviations from normality. Therefore, differences between anthropometric measurements performed in November 2018 and June 2022 were assessed by Students’ *t*-tests for dependent samples. In addition, Cohen’s *d* indicator was used to assess the effect size of these differences. The interpretation of Cohen’s *d* indicator for sports sciences is as follows [36]: trivial (<0.2), small (0.21–0.6), moderate (0.61–1.2), large (1.21–1.99), and very large (>2.0). The relationship between body composition vs. parametric and non-parametric variables was determined based on the Pearson product-moment correlation coefficient and the results of the chi-squared test, respectively. Cohen’s cutoff values were utilized to categorize the correlations: r < 0.1—very small, 0.1 ≤ r < 0.3—small, 0.3 ≤ r < 0.5—moderate, and r ≥ 0.5—large [37]. Statistical significance was set at *p* < 0.05.

## 3. Results

The training regimes and the demographic characteristics of Master swimmers are presented in Table 1.

The changes in the body composition and the sarcopenia indicators of Master swimmers over a 4-year period (November 2018—PRE to June 2022—POST) are presented in Table 2.

As shown in Table 2, none of the analyzed body composition parameters deviated significantly during the 4-year period. Sarcopenia indicators, i.e., Muscle Mass Index and Skeletal Muscle Index, did not change significantly during the analyzed period.

Correlations between the training intensity index and body composition parameters of the Master swimmers are presented in Table 3.

Inconsequential correlations were noted between training loads and body composition parameters in female athletes. However, during the second examination, the training intensity index was positively and significantly correlated with total body water, fat-free mass, skeletal muscle mass, basal metabolic rate, and sarcopenia indicators in male athletes (Table 3).

Sports experience was not correlated with any body composition parameters. Female and male participants with a history of SARS-CoV-2 infection did not differ from non-infected subjects in terms of body composition.

In the survey conducted in June 2022, 23 men (50%) and 6 women (42.8%) declared to have been infected with SARS-CoV-2 during the pandemic. The most frequently reported symptoms were decreased exercise tolerance (37%), headache (31%), arthritis pain (31%), olfactory and taste impairment (24%), and chronic cough (24%). Six percent of the examined athletes reported no symptoms accompanying the infection. The majority of infected athletes (62%) did not take any medications, whereas others took paracetamol (27%) and ibuprofen (11%). None of the examined athletes was hospitalized for COVID-19.

## 4. Discussion

The main finding of our study is that the four-year period covering the COVID-19 pandemic did not have an impact on body composition parameters in middle-aged Master swimmers. Moreover, participants with a history of SARS-CoV-2 infection did not differ notably in terms of body composition from those who did not suffer from infection. In fact, the participant’s body mass index (BMI) and body fat percentage were below the values recommended for healthy Caucasian women and men of the same age [38]. The waist-to-hip ratio was also within the ranges recommended by the World Health Organization [39]. However, data concerning time-related changes in the body composition of Master athletes are scarce. A ten-year study of Master cyclists did not reveal any associations between age, body mass, and BMI [40]. Before the pandemic, some studies revealed changes in body composition as a result of various training modes in untrained individuals aged 35 or older. For example, a 3-year observational study involving healthy and physically active older adults revealed no changes in total body weight but reported a decrease in fat-free soft tissue and skeletal muscle mass [41]. The cited authors concluded that leisure-time PA may not be sufficient to delay the negative changes in body composition in older adults. Other studies demonstrated changes in body composition in middle-aged and older adults after a short-term PA intervention. In a study of middle-aged, non-obese, untrained women and men, a 3-month aerobic training program led to changes in the subject’s body weight, body fat percentage, and BMI [42,43]. Regular and long-term aerobic exercise also influenced lean body mass in healthy women and men aged <65 [44]. In contrast, a 16-week water aerobics intervention program did not induce changes in the body mass, body fat mass, body fat percentage, and waist-to-hip ratio of middle-aged women (45 years of age) [45].

When analyzing the results of this study, it is important to bear in mind the COVID-19 pandemic context. The analyzed four-year period included the COVID-19 pandemic when access to sports and recreational activities was limited. All swimming pools were closed between March 2020 and May 2020. In the following months, local restrictions on the use of indoor swimming pools were imposed temporarily based on the local incidence rate of SARS-CoV-2 infection. During this period, swimming pools were accessible in certain parts of the country but closed in other regions. The mitigating strategies for swimming-related activities were lifted in the summer of 2021. The COVID-19 lockdown resulted in the suspension of sports and recreational activities for many weeks, which led to the atrophy and diminishing functionality of muscles, especially in untrained older adults [46]. However, BMI and body fat percentage did not change in young kayak athletes who performed regular aerobic and strength training at home during a seven-week lockdown [47]. The pandemic had no effect on the body composition of elite football players who trained at home [48]. In addition, no changes in body composition were observed in male adult futsal players who did not undertake regular PA during a 70-day lockdown quarantine [49].

In the current study, the examined swimmers did not train regularly at home during subsequent lockdowns, and their body composition parameters did not change markedly during the 4-year period. Moderate, positive correlations were found between training intensity index and skeletal muscle mass, as well as basal metabolic rate and sarcopenia indicators among male swimmers in 2022. Based on the data, we conclude that long-term swimming training, performed with proper physiological load, could delay negative age-related changes in body composition. It is plausible that the mild case of the SARS-CoV-2 infection in our swimmers was related to their low body fat parameters, as shown in the study conducted by Yoshiji et al. [50]. The study revealed an association between increased body fat mass, increased body fat percentage, and COVID-19 severity.

In the pre-pandemic era, Macek et al. [51] reported a systematic increase in body fat percentage and fat mass and a decrease in fat-free mass in middle-aged and older non-athlete adults assessed at 4-year intervals. No such changes were noted in the present study. This is most likely due to the fact that the Master swimmers were extensively involved in sports activity for many years before the lockdown. Apparently, training cessation during the pandemic did not have an effect on their body composition parameters. Moreover, the tested swimmers were characterized by a higher basal metabolic rate than sedentary adults of the same age [52]. According to the latest studies, a higher basal metabolic rate is a biomarker of healthy aging [53]. A comparison of the present findings with the results of a study conducted on a large cohort of healthy middle-aged women and men revealed that Master swimmers were characterized by lower values of BMI, body fat mass, waist-to-hip ratio, and body fat percentage than untrained peers [54,55]. Our study did not reveal sarcopenia risk in Master athletes. On both dates of examination, our male participants presented higher values of Muscle Mass Index than recommended by the European Working Group on Sarcopenia in Older People (i.e., above 10.7 kg/m^2^). A difference amongst genders in sarcopenia indicators was observed, as noted by Lee et al. [56], but values of the Muscle Mass Index in our female participants were also within the recommended range, i.e., above 6.76 kg/m^2^. Moreover, our female Master swimmers presented higher values concerning the Muscle Mass Index and Skeletal Muscle Index compared to healthy, normal-weight young women (9.5 kg/m^2^ vs. 8.7 kg/m^2^, and 41.8% vs. 40.3%, respectively) [57]. These observations indicate that swimming training practiced regularly for years can delay unfavorable changes in body composition in middle-aged subjects.

The present study includes certain limitations. Firstly, the number of participants examined in 2022 decreased markedly when compared to the baseline. More than one hundred and sixty Master swimmers were evaluated at baseline, but the final analysis involved only the results of sixty participants. Reasoning is yet to be determined as to why so many Master athletes did not participate in the most important national event of 2022. Secondly, we implemented guidelines contained in the European Working Group on Sarcopenia in Older People (EWGSOP1) instead of the newer version EWGSOP2. According to Wallengren et al. [58], there are minute differences between EWGSOP1 and 2, but they state that meaningful differences between EWGSOP1 and 2 in 85-year-olds cannot be ruled out. Since our study did not include any participants >80 years old, and our population consisted of normal walkers, this choice had an undetectable impact on the results. Thirdly, we did not analyze the differences between the body composition of women and men, as these differences have been confirmed in previous studies [59]. Lastly, the subjects’ physical activity was not monitored during subsequent lockdowns; therefore, the impact of physical activity (or inactivity) on body composition could not be determined. Despite this, the study provides reliable insights into the long-term effects of swimming training on the body composition of middle-aged athletes. Further research should involve a larger number of participants to confirm the findings of the current study. Furthermore, additional research could explore the liaisons between body composition and other lifestyle variables, e.g., diet, sleep, and current occupation.

## 5. Conclusions

Our study showed that a period of 4 years covering several COVID-19 lockdowns did not have an impact on body composition parameters in middle-aged Master swimmers. No significant changes in any of the body composition parameters were detected. Moreover, body composition parameters did not differ in participants with a history of SARS-CoV-2 infection when compared to the non-infected ones. Thus, the SARS-CoV-2 infection did not affect body composition parameters in assessed swimmers. Sarcopenia symptoms were not detected in examined athletes on both dates of examination. Based on the aforementioned observations, it can be concluded that regular swimming training appears to promote healthy aging. Further research involving a larger population is needed to validate these observations.

## Figures and Tables

**Table 1:** The participants’ training regime and demographic characteristics at baseline (mean values ± standard deviation).

Sex	Age [Years]	Body Height [cm]	Number of Training Sessions per Week	Distance Covered in Training Session [km]	Training Intensity Index	Sports Experience [Years]
Males (*n* = 46)	47.0 ± 11.0	180.2 ± 4.8	3.2 ± 1.1	2.8 ± 0.9	9.9 ± 5.3	17.0 ± 11.1
Females (*n* = 14)	47.2 ± 12.2	165.7 ± 8.3	3.0 ± 1.2	2.7 ± 0.8	8.6 ± 4.8	15.0 ± 7.5

**Table 2:** Body composition parameters and sarcopenia indicators of male and female Master swimmers measured in November 2018 (PRE) and June 2022 (POST) (mean values ± standard deviation).

		Males (*n* = 46)	*t*-Test *p* Value/ Cohen’s *d*	Females (*n* = 14)	*t*-Test *p*-Value/ Cohen’s *d*
Body mass [kg]	PRE	83.73 ± 8.48	*p* = 0.831 *d* = 0.010	62.49 ± 7.68	*p* = 0.593 *d* = 0.046
	POST	83.82 ± 9.04		62.84 ± 7.66	
Total body water [L]	PRE	51.08 ± 4.40	*p* = 0.632 *d* = 0.019	35.13 ± 4.64	*p* = 0.414 *d* = 0.044
	POST	51.17 ± 4.80		34.93 ± 4.36	
Body fat mass [kg]	PRE	14.13 ± 4.96	*p* = 0.945 *d* = 0.004	14.63 ± 5.32	*p* = 0.408 *d* = 0.115
	POST	14.11 ± 4.93		15.22 ± 4.98	
Fat free mass [kg]	PRE	69.59 ± 6.01	*p* = 0.636 *d* = 0.019	47.86 ± 6.32	*p* = 0.447 *d* = 0.041
	POST	69.71 ± 6.59		47.61 ± 5.96	
Skeletal muscle mass [kg]	PRE	39.66 ± 3.62	*p* = 0.672 *d* = 0.018	26.36 ± 3.81	*p* = 0.533 *d* = 0.032
	POST	39.73 ± 4.02		26.24 ± 3.63	
Body mass index [kg/m^2^]	PRE	25.74 ± 2.23	*p* = 0.833 *d* = 0.013	22.79 ± 2.50	*p* = 0.667 *d* = 0.041
	POST	25.77 ± 2.36		22.89 ± 2.35	
Percent body fat [%]	PRE	16.65 ± 4.58	*p* = 0.919 *d* = 0.000	23.18 ± 7.30	*p* = 0.347 *d* = 0.121
	POST	16.65 ± 4.54		24.01 ± 6.44	
Basal metabolic rate [kcal]	PRE	1873.13 ± 129.63	*p* = 0.649 *d* = 0.019	1403.36 ± 136.57	*p* = 0.503 *d* = 0.037
	POST	1875.65 ± 142.40		1398.43 ± 128.60	
Waist-to-hip ratio	PRE	0.870 ± 0.052	*p* = 0.075 *d* = 0.155	0.846 ± 0.052	*p* = 0.221 *d* = 0.367
	POST	0.878 ± 0.051		0.864 ± 0.046	
Visceral fat level	PRE	5.74 ± 2.42	*p* = 0.679 *d* = 0.025	5.86 ± 2.66	*p* = 0.362 *d* = 0.171
	POST	5.80 ± 2.34		6.29 ± 2.37	
Muscle Mass Index [kg/m^2^]	PRE	12.18 ± 0.75	*p* = 0.729	9.55 ± 0.69	*p* = 0.579
	POST	13.00 ± 0.86	*d* = 1.016	9.51 ± 0.64	*d* = 0.060
Skeletal Muscle Index [%]	PRE	47.48 ± 2.66	*p* = 0.938	42.27 ± 4.30	*p* = 0.370
	POST	47.49 ± 2.67	*d* = 0.004	41.83 ± 3.79	*d* = 0.109

**Table 3:** Pearson’s correlation coefficient between training intensity index and body composition parameters in male Masters swimmers assessed in November 2018 (PRE) and June 2022 (POST).

		Pearson’s Coefficient	*p*-Value
Body mass [kg]	PRE	0.16	0.290
	POST	0.15	0.329
Total body water [L]	PRE	0.27	0.065
	**POST**	**0.31**	**0.037**
Body fat mass [kg]	PRE	−0.06	0.678
	POST	−0.15	0.334
Fat-free mass [kg]	PRE	0.27	0.064
	**POST**	**0.31**	**0.037**
Skeletal muscle mass [kg]	PRE	0.28	0.057
	**POST**	**0.33**	**0.026**
Body mass index [kg/m^2^]	PRE	0.07	0.654
	POST	0.06	0.689
Percent body fat [%]	PRE	−0.14	0.366
	POST	−0.24	0.100
Basal metabolic rate [kcal]	PRE	0.27	0.064
	**POST**	**0.31**	**0.035**
Waist-to-hip ratio	PRE	−0.07	0.640
	POST	−0.10	0.513
Visceral fat level	PRE	−0.07	0.637
	POST	−0.14	0.340
Muscle Mass Index [kg/m^2^]	PRE	0.26	0.083
	**POST**	**0.33**	**0.024**
Skeletal Muscle Index [%]	PRE	0.17	0.258
	**POST**	**0.31**	**0.038**

## Data Availability

The datasets used and analyzed during the current study are available from the corresponding author upon reasonable request.
